# Novel Antimicrobials from Computational Modelling and Drug Repositioning: Potential *In Silico* Strategies to Increase Therapeutic Arsenal Against Antimicrobial Resistance

**DOI:** 10.3390/molecules30112303

**Published:** 2025-05-24

**Authors:** Antonio Tarín-Pelló, Sara Fernández-Álvarez, Beatriz Suay-García, María Teresa Pérez-Gracia

**Affiliations:** 1 Área de Microbiología, Departamento de Farmacia, Instituto de Ciencias Biomédicas, Facultad de Ciencias de la Salud, Universidad Cardenal Herrera-CEU, CEU Universities, Alfara del Patriarca, 46115 Valencia, Spain; antonio.tarin@alumnos.uchceu.es (A.T.-P.); sara.fernandezalvarez@alumnos.uchceu.es (S.F.-Á.); 2 ESI International Chair@CEU-UCH, Departamento de Matemáticas, Física y Ciencias Tecnológicas, Universidad Cardenal Herrera-CEU, CEU Universities, C/San Bartolomé 55, Alfara del Patriarca, 46115 Valencia, Spain; beatriz.suay@uchceu.es

**Keywords:** computational models, drug repositioning, antimicrobial resistance, mathematical prediction models, machine learning, deep learning, molecular docking, molecular dynamics, QSAR models, topological data analysis

## Abstract

Antimicrobial resistance (AMR) is one of the most significant public health threats today. The need for new antimicrobials against multidrug-resistant infections is growing. The development of computational models capable of predicting new drug–target interactions is an interesting strategy to reposition already known drugs into potential antimicrobials. The objective of this review was to compile the latest advances in the development of computational models capable of identifying drugs already registered by the Food and Drug Administration for other indications with potential capacity to be applied as antimicrobials. We present studies that apply *in silico* methods such as machine learning, molecular docking, molecular dynamics and deep learning. Some of these studies have *in vitro*/*in vivo* results that demonstrate the reliability of this computational methodology in terms of the identification of effective molecules and new targets of interest in the treatment of infections. In addition, we present the methods that are under development and their future prospects in terms of the search for new antimicrobials. We highlight the need to implement these strategies in the research of effective drugs in the treatment of infectious diseases and to continue to improve the available models and approaches to gain an advantage against the rapid emergence of AMR.

## 1. Introduction

Antimicrobial resistance (AMR) is one of today’s most important public health threats [1]. The most recent data on estimates associated with bacterial AMR assumed 4.71 million deaths in 2021, of which 1.14 million were directly attributable to AMR. In order to address this global health problem, different approaches aimed at treating multidrug-resistant infections have been studied in the current therapeutic arsenal. Examples include the application of monoclonal antibodies, nanoparticles, antimicrobial peptides and phage therapy, among others [2]. Although these approaches have shown some efficacy in terms of prevention and improved prognosis in infectious diseases, their clinical use remains complementary to traditional antimicrobial therapy, acting as antimicrobial adjuvants. Therefore, the search for new antimicrobials effective against multidrug-resistant microorganisms remains imperative.

**Figure 1:**
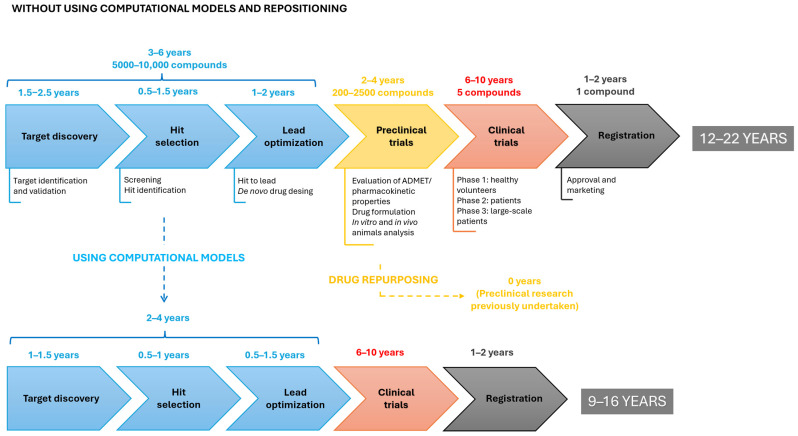
Duration of drug development from *de novo* synthesis compared to the use of computational methods and drug repositioning.

The emergence of the SARS-CoV-2 virus has accelerated the implementation of new strategies to find effective treatments [3]. Among these strategies is the development of mathematical predictive models. The development of computational models capable of predicting drugs and/or drug targets with potential antimicrobial capacity from biomedical datasets is a strategy increasingly used in preclinical trials. The methodology of these models allows for an acceleration of development times and, consequently, a reduction in the costs associated with these processes (Figure 1).

The application of computational methods such as machine learning (ML) or molecular docking (MD) is, according to recent studies, the future for the development of new drugs, including antimicrobials [4,5,6]. The potential of these bioinformatic techniques includes the ability to reposition drugs, i.e., to provide new therapeutic applications for drugs already used in the clinic for other diseases [7,8]. Among the drugs that can be detected by these prediction models are those that, despite being used in the clinic for non-infectious diseases, have a certain affinity for microbial targets [9]. It is also possible to find antimicrobials that are commonly indicated for certain microorganisms that, according to some models, would be able to broaden their spectrum.

The aim of this review is to analyse strategies, individually or in combination, that have predicted the possible repositioning of non-antimicrobial drugs to potential antimicrobials against certain infections and antimicrobials with the ability to broaden their spectrum.

## 2. Computational Strategies with the Ability to Predict Repositioning of Known Drugs to Antimicrobials

**Figure 2:**
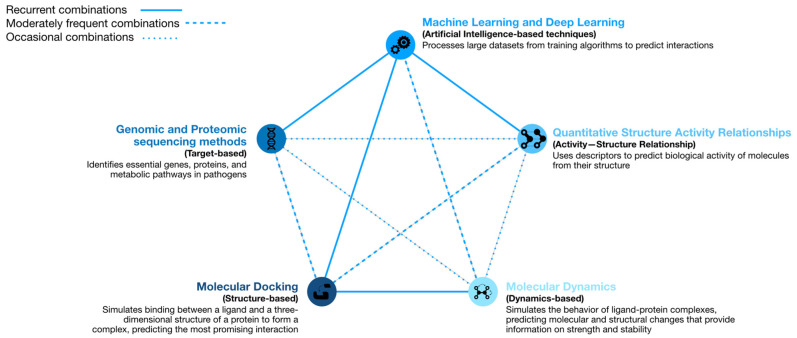
The main computational strategies for the discovery of new antimicrobials and their combinations. The combinations have been classified into 3 categories: the widely applied ones (solid line) are the most used because they are employed separately, and their results have a known criterion; the frequently applied ones (large dashed line), although they do not present as great a criterion as the previous ones, when combined, provide information that cannot be obtained with the separate methods; and the occasionally applied ones (small dashed line) are carried out for specific questions or needs of the study to be carried out (genomic data of interest or molecular structures that cannot be observed with more traditional computational models); however, they are young computational models with limited reliability.

Computational models can present different approaches within the development of new antimicrobials or the repositioning of non-antimicrobial drugs [10]. In this section, we present the most recent advances in computational strategies, such as ML, MD or systems biology, which have been able to identify drug candidates with potential efficacy for antimicrobial application. As the reading progresses, we will be able to observe different combinations of the models presented in this work. Figure 2 shows a summary of the combinations of models observed in the literature and their frequency.

### 2.1. Machine Learning

The application of artificial intelligence (AI) in computational models has proven to be very useful in the current era of Big Data [9]. The use of large biomedical databanks poses a difficulty in information management that is being solved by tools such as ML. We use ML to define any computational methodology that is capable of rapidly predicting drug–target interactions (DTI) by providing the model with data banks and an algorithm capable of processing them through training [11].

Model training is defined as the process by which a model is taught to make predictions with the input data. For proper training, algorithms are generally developed that are capable of processing data of interest previously obtained by other methods, such as *in vitro* assays. A clear example is the work of Shehadeh et al., in which they used the results of a high-throughput liquid infection assay by methicillin-resistant *Staphylococcus aureus* (MRSA) in a model of *Caenorhabditis elegans* to develop an ML model to predict compounds with antibiotic activity [12] (Table 1). The model identified among 22,768 compounds 45 molecules with potential antimicrobial activity that the first *in vitro* screen was unable to identify. These results translate into resource savings, as the model demonstrated a higher predictive efficiency than the high-throughput *in vitro* screening used as the first database. Another example of ML is the Condition-specific Antibiotic Regimen Assessment using Mechanistic Learning (CARAMeL) approach by Chung et al. where a flow-based model was designed to simulate the impact of pathogen metabolic heterogeneity on DTIs [13]. This is a model that applies pathogen chemogenomic and transcriptomic databanks to design condition-specific antibiotic regimens, thus predicting more specific treatments for each infection. In this study, they focused on the CARAMeL model with *Escherichia coli*, which was able to interpret metabolic issues and resistance mechanisms of this pathogen from the data provided, predicting a total of 24 effective combination therapies, some already in routine clinical use. These *in silico* predictions of combination therapies allow for the broadening of the spectrum of antibiotics already known, not only against bacteria, but also against fungi and some cancers [13].

### 2.2. Molecular Docking

MD is a computational tool capable of predicting the most promising interaction between a ligand and a three-dimensional protein structure to form a complex [14]. It is one of the most widely applied computational tools in drug discovery and development, both alone and in combination with other *in silico* tools. In this case, the MD analyses the interaction of drugs of interest against specific proteins of pathogenic microorganisms [15]. Therefore, this methodology requires a deep understanding of the bacterial proteome to identify promising targets, such as essential pathogen proteins or virulence factors involved in host colonisation or intracellular survival [16]. An example of this is the work by Madugula et al., which presented a polypharmacological approach with the aim of discovering antimycobacterial activity by performing high-throughput docking studies against 20 *M. tuberculosis* targets of interest [17]. These targets were extracted from the Molecular Property Diagnostic Suite–Tuberculosis library and were tested alongside 300 Food and Drug Administration (FDA)-approved drugs. Following MD and subsequent predictive activity spectra for substances (PASS) analysis, 34 drugs were identified as having antituberculosis activity, of which 21 were antibiotics, demonstrating the reliability of the *in silico* study. As for the rest of the drugs identified by the model, it was observed that they were in different stages of discovery for the treatment of different diseases, such as cancer, degenerative diseases and dengue virus infection, among others.

Many studies perform *in silico* methods prior to *in vitro* and *in vivo* assays. This is because they seek to apply computational tools as a first filter to reduce variables such as the number of drugs or proteins to be tested *in vitro* [18]. This order of procedures allows for an acceleration in each of the subsequent *in vitro* and *in vivo* assays and, consequently, a reduction in the costs associated with these processes. However, there are interesting studies where these tools are applied once the molecules of interest have been selected, confirming and identifying the reason for the antimicrobial activity observed *in vitro* or *in vivo*. An example of this is the work by Zhou et al. in which they searched for inhibitors of fungal phosphotidylserine synthase (Cho1), an enzyme with a crucial role in the virulence and viability of several pathogenic fungi [19]. After different *in vitro* assays, the authors identified seven compounds with the ability to inhibit the Cho1 enzyme of *Candida albicans*. It was via MD that the competition of serine and CBR-5884 with yeast Cho1 was observed. CBR-5884 is a preclinical drug with the ability to inhibit *de novo* serine synthesis in addition to inhibiting the proliferation of cancer cell lines, such as melanoma and breast cancer, which have a high propensity for serine synthesis [20]. In this work, CBR-5884 was shown to interact directly with two residues essential for Cho1 activity, inhibiting Cho1 by competing with serine. Using similar methodology, Shaikh et al. identified the possibility of repositioning ebselen against *Serratia marcescens* infections [21]. Although *in vitro* assays already demonstrated the antibacterial activity of ebselen against *S. marcescens*, a subsequent MD study identified the strong binding of ebselen to specific quorum sensing (QS) proteins through hydrogen bonding and aromatic interactions. These results showed not only the antibiotic capacity of ebselen but, more specifically, its antibiofilm capacity.

In addition to identifying drugs with intrinsic antimicrobial capabilities, MD is also able to predict the efficacy of certain molecules as antimicrobial adjuvants. A study of synergism between the antidepressant sertraline and *Cinnamomum verum* essential oil against *Candida* species and their biofilms identified the association of these compounds *in vitro* [22]. This synergy allows a large decrease in the amounts of both compounds compared to their individual use in the treatment of yeast infections. For *in silico* testing, the cytochrome P450-dependent lanosterol 14α-demethylase (CYP51) target of *C. albicans* was selected because of its involvement in ergosterol synthesis and its inhibition as a mechanism of action of several antifungal agents. After a search in the Protein Data Bank (PDB) database (5V5Z), the target was entered into the Maestro environment using the Protein Preparation Wizard. The main component of the essential oil ((E)-cinnamaldehyde) was also entered into Maestro, generating its three-dimensional SMILES chain structure using Open Babel. Once the structures were obtained, and using AutoDock 4.2.6, MD was performed, obtaining different poses modelled with the Lamarckian genetic algorithm (LGA). Finally, the model predicted the most plausible pose, being the binding of the compound to the heme group of the protein and the intervention of the iron atom in this binding. The results demonstrated not only the antifungal activity of the essential oil, but also the antimicrobial and adjuvant properties that antidepressants are capable of exhibiting [23,24,25]. Another example of post-*in vitro* MD application and drug replacement is the study by Yang et al. investigating the activity of the natural alkaloid harmaline to combat *Klebsiella pneumoniae* strains with tmexCD1-toprJ1 gene clusters, which are capable of expressing plasmid-mediated efflux pumps that confer resistance to multiple drugs, including tigecycline [26,27]. *In vitro* studies evaluating the synergism of harmaline and tigecycline against tmexCD1-toprJ1-positive and -negative *K. pneumoniae* strains showed that tmexCD1-toprJ1-positive strains had significantly enhanced tigecycline activity compared to tmexCD1-toprJ1-negative strains. Using AutoDock Vina (v1.1.2) software and the PDB database, MD assays were performed where harmaline was docked to the target structures. Visualisations using PyMOL and Discovery Studio 2020 Client of the complex confirmed, together with the specific binding site mutation, the interaction of harmaline to the TMexC1/TMexD1/TOprJ1 subunits predicted by MD. The *in silico* results defined that harmaline was able to alter the secondary structure of all three targets. Therefore, harmaline could be repositioned as an antibiotic adjuvant due to its ability to prevent the development of tigecycline resistance in tmexCD1-toprJ1-positive bacteria, disrupt the bacterial membrane and trigger the proton motive force, thereby inhibiting the activity of efflux pumps.

These examples, among many others, have demonstrated the ability of MD to confirm and understand the antimicrobial properties of non-antimicrobial drugs [28,29,30,31,32,33]. The ability of this strategy to individually reposition drugs has been a breakthrough in the investigation of potential new antimicrobials (Table 2). Importantly, despite its advantages, MD has limitations and requires experimental validation to confirm predicted interactions. In fact, there are studies in which the possibility of finding effective drugs by MD has been reported, yet the subsequent *in vitro* results do not correlate with the results observed *in silico* [34]. This is why many authors propose to validate and improve the accuracy of the results obtained using strategies that combine MD with other computational tools.

### 2.3. Molecular Dynamics

One of the most commonly used *in silico* methods complementary to MD is molecular dynamics [35]. It is a tool that provides insight into the strength and stability of DTIs by predicting molecular and structural changes that occur in these interactions under the influence of intermolecular and intramolecular forces [36].

There are interesting studies with drug repurposing approaches and the application of molecular dynamics. One example is the study by Ohra et al. that aimed to identify drugs with potential antimicrobial activity from a pharmacovigilance approach [37]. Using the OpenVigil 2.1 tool, they queried the FDA Adverse Event Reporting System (FAERS) database for drugs with antimicrobial potential based on known adverse events. This type of DTI, the adverse events recorded for each drug and the targets of *Pseudomonas aeruginosa, S. aureus* and *Streptococcus pneumoniae* were processed in the Maestro module for MD and Desmond for molecular dynamics. After performing both *in silico* processes, several drugs with the potential to be repositioned as antimicrobials were identified, such as the antihypertensives lisinopril, olmesartan and valsartan, the statin atorvastatin, the antidiabetic rosiglitazone and the smoking cessation drug varenicline. However, molecular dynamics results identified stability issues in the lisinopril molecule against the penicillin-binding protein 3 (PBP3) of *P. aeruginosa*.

In addition to the discovery of potential antibiotics against Gram-positive and Gram-negative bacteria [38], there are numerous studies that apply both MD and molecular dynamics to identify effective drugs against mycobacteria [39,40,41,42]. Studies such as those by Medha et al. and Ezquerra-Aznárez et al. address the repositioning of non-antibiotic drugs using *in silico* models with a similar common methodology; based on the three-dimensional structures of the *M. tuberculosis* proteins of interest and drugs obtained from different databases, MD simulations are performed to find out the DTIs with the most stable binding energies. To find the best orientation in the binding site of the *M. tuberculosis* proteins, they allowed the drug to adopt different conformations. Once these energies have been calculated and after appropriate processing of the obtained DTIs, molecular dynamics simulations were performed to check the stability of the most stable complex predicted by the software used. In other words, they record the movement and interactions of the target with the ligand over a given time to observe whether it remains stable in the active site, whether it changes position or whether the binding unravels. Using this methodology, DTIs capable of inhibiting the growth and virulence of *M. tuberculosis* from different proteins were identified.

It is also possible to find literature on models that apply molecular dynamics to predict molecules capable of repositioning to drugs for the treatment of yeast infections [43,44]. Borgio et al. and David et al. conducted studies focused on the repositioning of non-antifungal drugs capable of being applied against *Candida* yeast infections. Both studies present a similar methodology observed in previous studies (MD + subsequent molecular dynamics), with the difference that each extracted the molecules for the study from different databases: ZINC in the case of Borgio et al. and DrugBank in the case of David et al. It is probably due to this difference in consultation that each study ascertained the possible antifungal repositioning of different drugs applied in the clinic for other types of diseases (Table 3).

Most of the molecules listed in Table 3 correspond to non-antibiotic drugs that have demonstrated *in silico* and/or *in vitro* activity as potential antimicrobials. However, there are also studies that have demonstrated an interesting ability to broaden the antimicrobial spectrum of some drugs, potentially enriching the available therapeutic arsenal [40,41,43,45]. Research such as that conducted by Shailaja et al. supports the imperative need to broaden the spectrum of already known antimicrobials, in addition to identifying adjuvants capable of preserving the antimicrobial activity of the available therapeutic arsenal [38,46]. Thus, the authors performed a virtual screening in the ZINC database in order to reposition molecules for the treatment of infections caused by superbugs producing the New Delhi metallo-β-lactamase 1 (NDM-1) enzyme, an enzyme that allows bacteria to inactivate the entire arsenal of β-lactam antibiotics, including carbapenemics, and is also ineffective against all clinically available β-lactamase inhibitors. Applying MD, the retinoid adapalene was identified as having a stable DTI against the NDM-1 enzyme. Since these results had shown that adapalene interacted with key amino acid residues in the active site of NDM-1, molecular dynamics simulations were performed, demonstrating stable conformational dynamics. Subsequent *in vitro* assays demonstrated that adapalene was not only able to inhibit the NDM-1 enzyme but was also able to act as an antibiotic adjuvant in combination with the carbapenemic meropenem in clinical isolates of *E. coli* and *K. pneumoniae*. Therefore, computational models have proven to be able not only to predict drugs with potential antimicrobial activity but also molecules that can function as antimicrobial adjuvants. Examples of this are the results obtained by Narimisa et al., identifying the citrus flavonoid diosmin, commonly used to promote vascular health, as an adjuvant in combination with ceftazidime or ciprofloxacin for infections by persistent *Salmonella* Typhimurium strains, as well as synergies of these antibiotics combined with the β-lactam nafcillin [47]. Another drug that, despite its β-lactam structure, is applied as an antidiabetic is metformin, which has been shown in MD and/or molecular dynamics studies to have adjuvant properties against different Gram-positive and Gram-negative bacteria [46,48].

### 2.4. Genomic and Proteomic Sequencing Methods

The study of genes, proteins and metabolites has led to a greater understanding and analysis of cellular functionality. This great development in recent years has made it possible not only to identify, characterise and quantify the molecular biology of organisms, but also to make use of bioinformatics to transform all these data into valuable information [49,50]. The application of *in silico* models capable of managing omics data has allowed the identification of new drugs and the repositioning of some already known drugs, thanks to the determination of molecular mechanisms involved in the pathogenesis of infectious diseases, such as the survival and virulence of the pathogen or the mechanisms of host parasitism.

**Figure 3:**
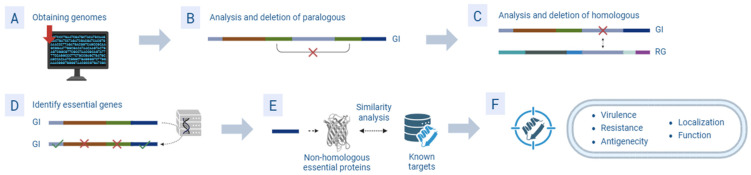
Graphical scheme of the steps commonly followed in the subtractive genomics approach. (**A**): Retrieval of interest and reference genomes from databases. (**B**): Removal of paralogous sequences in the genome of interest (GI). (**C**): Removal of homologous sequences to the reference genome (RG). (**D**): Compare non-homologous against database of essential genes to identify essential genes. (**E**): Identify and test for high similarity of non-homologous essential proteins to those found in databases of known targets. (**F**): Selection of targets of interest based on criteria of virulence, resistance, antigenicity, localization and function in the pathogen.

There are different *in silico* approaches based on omics analysis applied in microbiology. One of them is subtractive genomics, a technique that consists of identifying unique proteins necessary for the survival of bacteria [51] (Figure 3). These proteins are not present in the host, making them targets of interest to find drugs capable of interacting with them. A clear example of subtractive genomics is the study by Hassan et al., which aimed to search for therapeutic targets of *Shigella flexneri* serotype X and identify known drugs with the potential to interact with these targets [52]. To make these computational predictions, both the *S. flexneri* proteome and the human proteome were obtained from the Universal Protein Resource database. Both proteomes were compared using CD-HIT (v.2005) and BLAST (v.2.16.0) software to eliminate paralogous sequences and homologous proteins, obtaining 1803 non-homologous sequences between both proteomes. From these sequences, 1246 essential proteins were identified for *S. flexeneri*, which were compared with the DrugBank database for drug targets of interest and those drugs that interact with them. Following MD screening using Autodock software 4.2.6, the protein serine acetyltransferase was identified as a promising target for five compounds with antibacterial potential. These molecules corresponded to the antimigraine drug atogepant and the molecules olacaftor, fulacimstat, phthalocyanine and HQP1351, which are in clinical trials for the treatment of cystic fibrosis, cardiac pathologies and cancer, respectively. The results demonstrated how genomic sequencing methods can successfully identify effective and safe compounds against proteins related to virulence factors and antibiotic resistance in *S. flexneri*. This model also had an advantage over the identified targets, as the proteins were unique to the pathogen, avoiding possible adverse effects of the candidate molecules by not binding to human proteins, as these proteins did not show functional similarities to those of *S. flexneri.* The same approach was applied by Borges et al. in a chemogenomic model to reuse drugs already known as potential antibiotics against *Acinetobacter baumannii* [53]. For this purpose, a structural comparison between *A. baumannii* proteins and all drug targets present in the Therapeutic Targets Database and DrugBank databases was performed using the OrthoVenn2 and BLASTp servers. After extensive computational screening, including MD, 31 drugs that interacted with 14 proteins essential for *A. baumannii* were identified. These included the antifungal drug tavaborole, the antiparasitic drugs atovaquone and thioabendazole, the antitumour drugs homoharringtonine and MKT-077, the antirheumatic leflunomide, and the antiviral hepatitis C drug ribavirin. These seven drugs were tested *in vitro*, not only demonstrating their effectiveness as antibacterials, but also confirming the relevance of the bacterial targets predicted by the model as being of interest for the discovery of new antimicrobials, as they turned out to be proteins present in different metabolic pathways of interest for the survival, virulence and resistance of *A. baumannii*. Due to the results obtained, the authors did not rule out the possibility of the antimicrobial potential of the rest of the molecules predicted by the model, in addition to proposing *in vivo* assays to confirm the antibiotic efficacy of the molecules tested. Using a similar methodology, Santo et al. searched for new antifungals effective against sporotrichosis [54]. Similar to Hassan et al. and Borges et al., who studied *A. baumannii,* the model was able to predict molecules with activity against *Sporothrix brasiliensis,* such as the antitumour everolimus and the antifungal indicated for dermatomycosis bifonazole. Subsequent *in vitro* assays confirmed the predictions of the model, with both drugs demonstrating satisfactory minimum inhibitory concentration values. In addition, MD studies demonstrated the affinity of everolimus and bifonazole for the sterol-14-α-demethylase and serine/threonine-protein kinase TOR proteins, respectively.

A further step in *in silico* methodologies in this category is found in subtractive proteomics models, which identify essential proteins as relevant drug targets by screening the proteome of the organism of interest [55]. As these are essential proteins, screening is limited to only those targets that are of great importance for the survival of the microorganism under study. A study by Urra et al. applied this computational strategy to test whether it was possible to reposition known drugs as antibiotics against *P. aeruginosa* [56]. In this study, subtractive proteomics was applied to select proteins from a dataset of the *P. aeruginosa* proteome to be compared to the human genome. The screening consisted of discarding the proteins similar to both the microorganism itself and the ones from humans, as well as those similar to the human gut microbiota. As we have observed in previous studies, this procedure allows the elimination of unwanted DTIs for the drugs predicted by the model, thus avoiding adverse effects [51,52,53]. Finally, the proteins selected by the model were those belonging to the resistance/nodulation/cell division (RND) family used by *P. aeruginosa* as efflux pumps for antibiotics. A peculiarity of this study is that it was not possible to obtain *in vitro* data of the bacterial proteins present in the study, so a deep learning (DL) algorithm was integrated to predict the three-dimensional structure of the proteins from the databases. In addition, molecular dynamics simulations were performed using the Schrodinger Master environment in order to refine and improve the stability of the predicted three-dimensional structures. Finally, MD allowed us to consider different conformational states of proteins and to improve the identification of promising drugs. Among these were clinical-stage drugs such as the antimigraine drug MK-3207 and the antitumour drugs bemcentinib and suramin, which are promising candidates to combat *P. aeruginosa* infections [56].

The studies presented in this section demonstrate the importance of performing genomic and proteomic studies to gain a more realistic understanding of the DTIs that can occur in reality (Table 4). Transcriptomic studies are also of interest, as different tissue types, cellular conditions and environmental factors make the transcriptome dynamic in nature [57]. There are studies, such as the one by Das et al., where, using an *in silico* transcriptomic approach, it was possible to predict effective drugs against *S. aureus* infections [58]. For this, a transcriptomic analysis was performed at the retinal level of a mouse model infected with *S. aureus* to understand and determine the alterations and gene expressions of the bacteria in this infection model. Based on a systems biology approach, a connectivity map was made using the GeneMANIA package (v3.3.5) of Cytoscape software (v3.9.0) to identify three drugs with potential antibiotic activity. These were the hypoglycaemic drug glibenclamide, the experimental antiarrhythmic clofilium tosylate and the antimicrobial dequalinium, which is indicated for the treatment of some bacterial vaginosis and oral infections. Subsequent *in vitro* trials demonstrated the efficacy of all three drugs, both individually and in combination with each other, improving infection and inflammation caused by the host immune system, along with retinal function. Therefore, the model proved to be able to identify drugs with the potential to prevent and treat endophthalmitis caused by *S. aureus*.

### 2.5. Quantitative Structure–Activity Relationship Models

Quantitative Structure–Activity Relationship (QSAR) models have gained attention in the biomedical world for drug discovery, design and development [59,60,61]. This is because this methodology has provided remarkable time and resource savings compared to other drug development methods, such as *de novo* synthesis.

QSAR models use physicochemical, biological and toxicological descriptors to predict the biological activity of molecules from their structure [62]. It is through this quantitative and interdisciplinary analysis that the model is able to identify important determinants for the potential antimicrobial activities that a molecule may have. This methodology not only makes it possible to search for known drugs and reposition them as antimicrobials, but also, based on the identified determinants, it is possible to design new drugs with superior efficacy to those already known [63]. Therefore, QSAR models have laid the foundation for the design and development of new molecules with *in silico* pharmacological activity.

In the most recent literature, we can find some studies focused on the search for new antimicrobials that apply QSAR methods (Table 5). One example is the work by Kamble et al., who evaluated the efficacy of a battery of 148 compounds against the schizont stage of the parasite *Theileria annulata* [64]. These compounds were obtained from the Cayman epigenetic library and have now been approved by the FDA for cancer therapy, although they are still under investigation in clinical trials [65]. Following the *in vitro* assays required to demonstrate growth inhibition and parasite susceptibility to the compounds, seven drugs with potent activity against *T. annulata*-infected cells were identified. The compounds in question were SAHA, ryuvidine, BVT-948, TCE-5003, trichostatin A, methylstat and plumbagin. Once the drugs with the highest activity were identified, the binding of these molecules to their respective targets in *T. annulata* was investigated *in silico*. Due to the lack of three-dimensional structures of the targets, homology modelling from human target orthologues in the parasite was performed to generate reliable protein structures for the MD. Docking showed that all compounds exhibited high binding energies to their respective targets, highlighting SAHA, Trichostatin A and BVT-948 as potential leads for the development of new antiparasitics.

However, we have recently observed a reduction in the number of articles applying this *in silico* methodology for drug repositioning, which is more commonly used for *de novo* development or for the development of derivatives from known leads [59,62]. This is because there are more up-to-date models, some of them with a QSAR approach, that have shown greater efficiency in their *in silico* results compared to conventional models.

## 3. Future Directions

We have observed throughout this review that the more classical models are still able to provide new insights into molecules and targets of interest in the fight against AMR. However, the rate at which AMR emerges continues to be much faster than the rate at which we achieve results in the laboratory or from computational models [66]. Therefore, the development of new *in silico* models and the updating of known models could provide faster and more accurate results, resulting in a higher success rate in the identification of effective molecules against resistant infections.

One of the strategies that works best, and which we have mentioned in the previous sections, when it comes to discovering new models, is the combination of existing models. A new combination of computational tools is presented in the study by Ngidi et al., who combine virtual screening, MD and molecular dynamics with the SwissADME tool, which allows predictions to be made from a drug’s absorption, distribution, metabolism and excretion (ADME) data [51]. These data are essential for drug approval, as they reveal drug similarity to other drugs and bioavailability by estimating physicochemical properties. Using this model combination, the authors identified the anti-asthmatic zafirlukast as a potential antibiotic against *M. tuberculosis* infections. Another example of a novel model combination is by Gohain et al., who combined a subtractive genomics model with MD, molecular dynamics, ADMET (ADME + toxicity) predictions and Density Functional Theory (DFT) to identify potential inhibitors against *S. pneumoniae* [67]. Density Functional Theory analyses properties such as electronic affinities, ionisation potentials, orbital energies and molecular structures. Using this combination of computational tools, the non-steroidal anti-inflammatory drug bromfenac was identified as a potential antibiotic, which showed a higher affinity for the SigA sigma factor of *S. pneumoniae* RNA polymerase compared to the antibiotic ceftibuten.

Very recent models have proven to be effective predictors of effective antibiotics. In the 2000s, topological data analysis (TDA), a model based on algebraic topology and computational geometry capable of extracting qualitative information from available molecular databases, emerged as an evolution of QSAR models [68,69]. This methodology was able to reposition drugs against SARS-CoV-2, and recently was able to do the same against *E. coli*, for which antipsychotics, antidepressants, antitumour drugs, retinoids and more therapeutic groups with antibiotic potential against this bacterium were identified [46,70].

However, authors such as Suay-García et al. propose that TDA in combination with other computational models, such as DL, could greatly improve the design and development of new antimicrobials [71]. DL is a subset of ML where large datasets are fed together with complex algorithms that help machines and models learn by training without being explicitly programmed [72]. Although it is a relatively novel tool, it has been shown to be able to identify drugs that can be repositioned for the treatment of bacterial infections. A study by Joshi et al. applied DL regression algorithms to screen and identify FDA-approved drugs that could inhibit dihydrofolate reductase (DHFR) of *Salmonella* Typhi [73]. After applying DL, a total of 500 molecules were screened for the desired property. In order to refine the results of the study, an MD screen and a subsequent molecular dynamics screen were included. This combination of strategies resulted in four potential compounds to inhibit the DHFR enzyme: the antitumour drugs duvelisib and nilotinib, the anti-herpetic amenamevir, which is still in phase 3 clinical trials, and the dry eye syndrome drug lifitegrast. The molecular dynamics results concluded the usefulness of these drugs as potential treatments against typhoid fever by intervening in the function of the DHFR enzyme in *S.* Typhi.

The combination of TDA and DL, called topological deep learning (TDL), could significantly increase the type and amount of information to be obtained from biomedical databanks and improve the efficiency and robustness of their processing. Recent studies, such as those by Chen et al. and Hou et al., have demonstrated the accuracy of tools applying TDL for vaccine, antibody and antibiotic prediction [74,75]. However, the design of these tools still needs to be fine-tuned to improve the viability and affinity of the molecules to be predicted.

## 4. Conclusions

The progressive emergence of AMR to the available therapeutic arsenal exposes an immediate need for the search and development of new antimicrobials. Gaining new insights into targets of interest, along with antimicrobial drug repurposing, remains a priority task to gain an advantage over the speed at which microorganisms gain resistance to known antimicrobials. Computational models have demonstrated their ability to accelerate the processes involved in drug development and repositioning, providing new insights into drug targets of interest and potentially effective antimicrobials. These models are continuously evolving, either by training existing models or by creating new ones. However, the improvement and development of new models and approaches are still needed to further accelerate this race against time against the global health threat posed by AMR.

## Figures and Tables

**Table 1:** Drugs predicted as potential antibiotics with ML models.

Method	Predictions	ML Algorithm	Accurate Predictions	Reference
Mol2vec model (Morgan algorithm)	Machine learning-assisted high-throughput screening of low-molecular-weight molecules.	Balanced random forest classifier to predict molecules for anti-MRSA compounds.	AUC ^2^ of 0.795 with a sensitivity of 81% and a specificity of 70%.	[12]
CARAMeL	Simulating metabolic flux data using GEMs ^1^ and developing an ML model to predict combination therapy outcomes using flux from GEMs ^1^. Impact of pathogen metabolic heterogeneity on drug–target interactions predictions.	Random forest to predict combination therapy outcomes for *E. coli* and *Mycobacterium tuberculosis.*	AUROC ^3^ = 0.83 for synergy, AUROC ^3^ = 0.98 for antagonism.	[13]

^1^ GEMs: genome-scale metabolic models; ^2^ AUC: area under the curve; ^3^ AUROC: area under the receiver operating curve.

**Table 2:** Drugs repositioned as antimicrobials by MD.

Molecules	Class of Drug	Known Target	New Target	New Indication Predicted	Reference
Promethazine	First-generation antihistamine	Histamine H1, H2, NMDA, muscarinic, alpha-adrenergic and dopamine receptors; purinoceptors; voltage-gated potassium or sodium channel; calmodulin	Quorum sensing (proteins btaR1, btaR2 and btaR3) of *Burkholderia thailandensis*	Biofilm formation inhibition and lipase activity by suppression of quorum sensing of *B. thailandensis*	[28]
Derivates of entinostat	Antitumorals	Human histone deacetylase	Histone deacetylase of *M. tuberculosis*	Metabolism inhibitors, antimicrobial peptides promoters and rifampicin adjuvants against *M. tuberculosis*	[29]
Nitrofural	Antibiotic, treatment of trypanosomiasis	Glutathione reductase	Proteins 1BVR, 1P9L, 1W66, 1XFC, 1U2Q, 1YLK, 1ZAU, 2FUM, 2CIN, 2WGE, 2A86,2JCV, 2A5V, 2QO1, 2QKX, 1E9X, 1W2G and 1EYE of *M. tuberculosis*	Antimicobacterial and antitubercular	[17]
Stavudine	Antiretroviral	Reverse transcriptase	1BVR, 1P9L, 1XFC, 1U2Q, 1ZAU, 2FUM, 2CIN, 2WGE, 2A86, 2JCV, 2QO1, 2QKX, 1E9X and 1W2G of *M. tuberculosis*		
Quinine	Antiparasitic	Protoporphyrin IX of *Plasmodium falciparum*	Proteins 1BVR, 1DF7, 1P9L, 1XFC, 1U2Q, 4FDO, 1ZAU, 2FUM, 2CIN, 2WGE, 2A86, 2JCV, 2A5V, 2QO1, 2QKX, 1E9X and 1W2G of *M. tuberculosis*		
Quinidine	Antiparasitic Antiarrhythmic	Sodium channel	Proteins 1BVR, 1DF7, 1P9L, 1XFC, 1U2Q, 1ZAU, 2FUM, 2CIN, 2WGE, 2A86, 2JCV, 2A5V, 2QO1, 2QKX, 1E9X and 1W2G of *M. tuberculosis*		
Amlodipine	Calcium channel blocker. Antihypertensive	Voltage-dependent calcium channel	RNA polymerase β’subunit (RpoC) of *Streptococcus pyogenes*	Inhibition of RpoC of *S. pyogenes*	[30]
Ranitidine	Histamine H2 antagonist	Histamine H2 receptors			
Floxuridine	Antitumoral	Riboside phosphorylase, thymidylate synthetase	SLY gene, sly, fabps, gap and ef genes of *Streptococcus suis*	Hemolytic activity and expression levels of virulence-related genes of *S. suis*	[31]
Atovaquone	Antipaludic	Cytochrome bc1 complex and dihydroorotate dehydrogenase	FtsZ protein	Inhibition of FTsZ of *M. tuberculosis*	[34]
Paroxetine	Selective serotonin reuptake inhibitor	5-HT reuptake transporter			
Nebivolol	Antihypertensive	Beta-1 adrenergic receptor			
Atosiban	Inhibitor of oxytocin and vasopressin Delays preterm birth in pregnancy	Oxytocin receptors	Enzyme HemD	Inhibition of HemD of *M. tuberculosis*	[32]
Rutin	Flavonoid, vitamin supplement	Aldo-keto reductase and carbonyl reductase			
Disulfiram	Treatment of alcohol dependence	Dopamine beta-hydroxylase and aldehyde dehydrogenase, mitochondrial	Aldehyde dehydrogenase of *Cryptococcus neoformans*	Inhibition of aldehyde dehydrogenase of *C. neoformans*	[33]

**Table 3:** Drugs identified as potential antimicrobials from molecular dynamics.

Molecules	Class of Drug	New Indication Predicted	Docking Score	Binding Score (Kcal/mol)	References
Lisinopril	Antihypertensive	Inhibition of: 3-deoxy-manno-octulosonate cytidylyltransferase UDP-2,3-diacylglucosamine hydrolase PBP3 ^1^ of *P. aeruginosa*	−10.8 −9.2 −9.4	−89.3 −50.7 −70.6	[37]
Olmesartan	Antihypertensive	Inhibition of lipotheichoic acids flippase LtaA of *S. aureus*	−9.0	−75.4	
Atorvastatin	Lipid-lowering drug, statin		−8.6	−96.9	
		Inhibition of CDP-activated ribitol for teichoic acid precursors of *S. pneumoniae*	−7.4	−74.6	
Rosiglitazone	Antidiabetic	Inhibition of d-alanine ligase of *S. aureus*	−7.3	−70.4	
Varenicline	Aid in smoking cessation		−7.1	−48.7	
Valsartan	Antihypertensive	Inhibition of peptidoglycan deacetylase of *S. pneumoniae*	−7.4	−62.6	
Verapamil	Antihypertensive	Inhibition of protein PE_PGRS45 of *M. tuberculosis*	−6.2 to −5.9	−58.8	[39]
Entacapone Tolcapone	Treatment of Parkinson’s disease		−7.3 to −6.3 −7.9 to −6.3	−40.0 −39.3	
Dutasteride	Antiandrogenic. Treatment of prostate cancer	Inhibition of 1,3-β-glucanosyltranferase from *Candida auris*	-	≤−10	[43]
Digoxin	Cardiac glycoside, treatment of heart failure				
Ergotamine	Vasoconstrictor, treatment of cluster headaches and migraines				
Paritaprevir	Antiviral, treatment of infections caused by the hepatitis C virus				
Acarbose	Hypoglycemic	Inhibition of alfa-glucosidase of *C. albicans*	−11.5	-	[44]
Adapalene	Treatment of acne, retinoid	Inhibition of NDM-1 ^2^ enzyme of *E. coli* and *K. pneumoniae* alone or in combination with meropenem	-	−9.2	[38]
Selamectin	Parasiticide and antihelminthic in veterinary medicine	Inhibition of DprE1 enzyme of *M. tuberculosis.* Possible multitarget antibacterial compound	-	-	[40]
Accolate	Prophylaxis and treatment of asthma	Inhibition of FadD32 protein of *M. tuberculosis*	−9.3	−45.1	[41]
Sorafenib	Antitumoral		−10.0	−32.7	
Mefloquine	Antimalarial		−8.0	−26.8	
Loperamide	Antidiarrheal		−8.5	−21.5	
Phytochemicals of *Withania somnifera*	Complement in anti-inflammatory, antidiabetic, antimicrobial, analgesic, antitumoral, anti-stress, neuroprotective, cardioprotective, rejuvenating and immunomodulatory treatments	Inhibition PyrG protein of *M. tuberculosis*	−12.6 to −10.8	-	[45]
Glimepiride	Hypoglycemic	Inhibition of Tap protein of *M. tuberculosis*	−9.7	−51.9	[42]
Flecainide	Antiarrhythmic agent		−9.1	−44.6	
Flupirtine	Investigated for treatment of fibromyalgia		−8.9	−46.4	
Nimodipine	Calcium channel blocker, improvement in neurological outcomes		−7.0	−46.1	
Amlodipine	Calcium channel blocker, antihypertensive		−7.2	−42.6	

^1^ PBP3: penicillin-binding protein 3; ^2^ NDM-1: New Delhi metallo-β-lactamase 1.

**Table 4:** Drugs identified as potential antimicrobials from genomic and proteomic sequencing methods.

Molecules	Class of Drug	New Indication Predicted	Reference
Decitabine	Antitumoral, pyrimidine nucleoside analogue	Inhibition of phospho-2-dehydro-3-deoxyheptonate aldolase of *Gardnerella vaginalis*	[51]
Nitroglycerin	Nitrate vasodilator, preventive of different cardiac and circulatory problems		
Phthalocyanine	Tetrapyrrole fundamental parent, under investigation in clinical trial for its antitumoral and antifungal effects and treatment of different skin diseases	Inhibition of serine acetyltransferase of *S. flexneri* serotype X	[52]
Fulacimstat	Chymase inhibitor, under investigation in clinical trial for treatment of heart diseases and diabetic kidney disease		
Atogepant	Antimigraine, receptor for different molecules mediated by G proteins		
Olverembatinib	Bcr-Abl inhibitor, under investigation in clinical trial for treatment of different leukemias and gastrointestinal stromal tumours		
Olacaftor	Cystic fibrosis transmembrane conductance regulator, under investigation in clinical trial for treatment of cystic fibrosis		
Tavaborole	Antifungal, treatment of onychomycosis caused by dermatophytes	Inhibition of LeuRS of *A. baumannii*	[53]
Ribavirin	Antiviral, treatment of infections caused by hepatitis C virus	Inhibition of inosine 5′-phosphate dehydrogenase of *A. baumannii*	
Leflunomide	Immunomodulator, treatment of rheumatoid arthritis	Interaction with dihydroorotate dehydrogenase of *A. baumannii*	
Atovaquone	Antiparasitic, treatment of malaria and AIDS-associated diseases		
Homoharringtonine	Antitumoral, treatment of different leukemias	Inhibition of the 50S ribosomal subunit of *A. baumannii*	
Thiabendazole	Anthelmintic, tubulin inhibitor	Inhibition of succinate dehydrogenase of *A. baumannii*	
MKT-077	Antitumoral, inhibitor of mitochondrial hsp 70 family member.	Inhibition of chaperone DnaK of *A. baumanni*	
Bifonazol	Antifungal, treatment of fungal skin infections, such as dermatomycosis	Interaction with sterol-14-alfa-demethylase of *S. brasiliensis*	[54]
Everolimus	Antitumoral, inhibition of mammalian target of rapamycin (mTOR) kinase, prevention of organ transplant rejection and treatment of various malignancies	Interaction with serine/threonine-protein kinase TOR of *S. brasiliensis*	
Quercetin	Flavonoid, antioxidant with specific inhibition of quinone reductase (QR2)	Inhibition of MurG of *S. aureus.*	[55]
MK-3207	Antagonist of the calcitonin gene-related peptide type 1 receptor in humans, under investigation in clinical trial for migraine disorders	Inhibition of RND efflux pumps of *P. aeruginosa*	[56]
Bemcentinib (R-428)	Tyrosine-protein kinase receptor inhibitor, under investigation in clinical trial for myelodysplastic syndrome, melanoma, acute myeloid leukaemia, and mesothelioma		
Suramin	MFP protein inhibitor, under investigation in clinical trial for non-small cell lung carcinoma, prostate adenocarcinoma, autism spectrum disorder and acute kidney injury		
Glibenclamide	Hypoglycemic drugs in the treatment of non-insulin-dependent diabetes mellitus	Reverse the expression of the master regulators perturbed in *S. aureus* endophthalmitis	[58]
Clofilium tosylate	Benzene, under investigation in clinical trial for heart rhythm disorders		
Dequalinium (fluomizin)	Antimicrobial, treatment of vaginosis and oral infections		

**Table 5:** Drugs identified as potential antimicrobials from Quantitative Structure–Activity Relationship models.

Molecules	Class of Drug	Score		New Indication Predicted	References
C_19_H_14_N_6_S, C_19_H_14_N_6_OS, C_19_H_13_FN_6_S, C_18_H_14_N_6_O_2_S, C_18_H_14_N_6_O_3_S.	Pyridothienopyrimidine derivatives, no previous known pharmacological activity	pMIC ^1^ −1.5 −1.2 −1.6 0.4 0.8		Inhibition of *P. aeruginosa* growth, unknown mechanism of action	[59]
C_23_H_21_N_4_Cl_3_O_2_S	Indazole compounds	p*K*_i_^2^ 2.8		Inhibition of S-adenosyl homocysteine/methylthio-adenosine nucleosidase (SAH/MTAN) of *E. coli* mediated quorum sensing to produce AMR	[61]
Sigmacidins (C_21_H_13_N_2_Cl_3_O_4_S)	Benzoic acid derivatives, no previous known pharmacological activity	Experimental pMIC ^1^: 5.7 2D QSAR pMIC ^1^: 4.9 3D QSAR pMIC ^1^: 5.2		Inhibition of bacterial RNA polymerase-σ factor interaction of *Streptococci*/*S. pneumoniae*	[62]
SAHA	Anti-cancer histone deacetylase inhibitor	ADME properties ^3^ within the margins	No Toxicity ^3^	Inhibition of epigenetic pathways of *T. annulata*-infected cells	[64]
Trichostatin A					
BVT-948,	PRMT inhibitor		No Toxicity ^3^		
TCE-5003			Hepatotoxicity ^3^		
Methylstat	Histone demethylase inhibitor		Hepatotoxicity ^3^		
Plumbagin	ROS/apoptosis inducer inhibitor		AMES toxicity ^3^		

^1^ pMIC: negative algorithm of Minimum Inhibitory Concentrations; ^2^ p*K*_i_: negative algorithm of *K*_i_, an evaluator of the direct interaction between an inhibitor and its molecular target; ^3^ Parameters calculated from QikProp: absorption, distribution, metabolism and excretion (ADME). Parameters calculated from pkCSM: toxicity.

## Data Availability

No new data were created or analysed in this study. Data sharing is not applicable to this article.

## Abbreviations

The following abbreviations are used in this manuscript:

ADME	Absorption, distribution, metabolism and excretion
ADMET	ADME + toxicity
AI	Artificial intelligence
AMR	Antimicrobial resistance
AUC	Area under the curve
AUROC	Area under the receiver operating curve
CARAMeL	Condition-specific Antibiotic Regimen Assessment using Mechanistic Learning
Cho1	Fungal phosphatidylserine synthase
CYP51	Sterol 14-demethylase
DFT	Density Functional Theory
DHFR	Dihydrofolate reductase
DL	Deep learning
DTI	Drug–target interactions
FAERS	FDA Adverse Event Reporting System
FDA	Food and Drug Administration
GEMs	Genome-scale metabolic models
LGA	Lamarckian genetic algorithm
MD	Molecular docking
ML	Machine learning
MRSA	Methicillin-resistant *Staphylococcus aureus*
NDM-1	New Delhi metallo-β-lactamase
PASS	Prediction of activity spectra for substance
PBP3	Penicillin-binding protein 3
PDB	Protein Data Bank
QS	Quorum sensing
QSAR	Quantitative Structure–Activity Relationship
RND	Resistance nodulation division
TDA	Topological data analysis
TDL	Topological deep learning
